# Association of Shelter-in-Place Hotels With Health Services Use Among People Experiencing Homelessness During the COVID-19 Pandemic

**DOI:** 10.1001/jamanetworkopen.2022.23891

**Published:** 2022-07-27

**Authors:** Mark D. Fleming, Jennifer L. Evans, Dave Graham-Squire, Caroline Cawley, Hemal K. Kanzaria, Margot B. Kushel, Maria C. Raven

**Affiliations:** 1 School of Public Health, University of California, Berkeley; 2 Benioff Homelessness and Housing Initiative, University of California, San Francisco; 3 Center for Vulnerable Populations, Department of Medicine, University of California, San Francisco; 4 Department of Emergency Medicine, University of California, San Francisco; 5 Phillip R. Lee Institute for Health Policy Studies, University of California, San Francisco

## Abstract

**Importance:**

Some jurisdictions used hotels to provide emergency noncongregate shelter and support services to reduce the risk of COVID-19 infection among people experiencing homelessness (PEH). A subset of these shelter-in-place (SIP) hotel guests were high users of acute health services, and the association of hotel placement with their service use remains unknown.

**Objective:**

To evaluate the association of SIP hotel placements with health services use among a subset of PEH with prior high acute health service use.

**Design, Setting, and Participants:**

This study used a matched retrospective cohort design comparing health services use between PEH with prior high service use who did and did not receive a SIP hotel placement, from April 2020 to April 2021. The setting was 25 SIP hotels in San Francisco, California, with a daily capacity of 2500 people. Participants included PEH who were among the top 10% high users of acute medical, mental health, and substance use services and who had 3 or more emergency department (ED) visits in the 9 months before the implementation of the SIP hotel program. Data analysis for this study was performed from February 2021 to May 2022.

**Exposures:**

SIP hotel placement with on-site supportive services.

**Main Outcomes and Measures:**

The primary outcomes were ED visits, hospitalizations and bed days, psychiatric emergency visits, psychiatric hospitalizations, outpatient mental health and substance use visits, and outpatient medical visits.

**Results:**

Of 2524 SIP guests with a minimum of 90-day stays, 343 (13.6%) met criteria for high service use. Of 686 participants with high service use (343 SIP group; 343 control), the median (IQR) age was 54 (43-61) years, 485 (70.7%) were male, 283 (41.3%) were Black, and 337 (49.1%) were homeless for more than 10 years. The mean number of ED visits decreased significantly in the high-user SIP group (1.84 visits [95% CI, 1.52-2.17 visits] in the 90 days before SIP placement to 0.82 visits [95% CI, 0.66-0.99 visits] in the 90 days after SIP placement) compared with high-user controls (decrease from 1.33 visits [95% CI, 1.39-1.58 visits] to 1.00 visits [95% CI, 0.80-1.20 visits]) (incidence rate ratio [IRR], 0.60; 95% CI, 0.47-0.75; *P* < .001). The mean number of hospitalizations decreased significantly from 0.41 (95% CI, 0.30-0.51) to 0.14 (95% CI, 0.09-0.19) for SIP guests vs 0.27 (95% CI, 0.19-0.34) to 0.22 (95% CI, 0.15-0.29) for controls (IRR, 0.41; 95% CI, 0.27-063; *P* < .001). Inpatient hospital days decreased significantly from a mean of 4.00 (95% CI, 2.44-5.56) to 0.81 (95% CI, 0.40-1.23) for SIP guests vs 2.27 (95% CI, 1.27-3.27) to 1.85 (95% CI, 1.06-2.65) for controls (IRR, 0.25; 95% CI, 0.12-0.54; *P* < .001), as did psychiatric emergency visits, from a mean of 0.03 (95% CI, 0.01-0.05) to 0.01 (95% CI, 0.00-0.01) visits for SIP guests vs no change in the control group (IRR, 0.25; 95% CI, 0.11-0.51; *P* < .001).

**Conclusions and Relevance:**

These findings suggest that in a population of PEH with high use of acute health services, SIP hotel placement was associated with significantly reduced acute care use compared with high users without a placement.

## Key Points

**Question:**

Was placement in a shelter-in-place (SIP) hotel during the COVID-19 pandemic associated with health system utilization among people experiencing homelessness with a history of high use of acute health services?

**Findings:**

In this cohort study of 686 high users of acute county services experiencing homelessness, those who received a SIP hotel placement had significantly fewer emergency department visits, hospital admissions, inpatient days, and psychiatric emergency department visits compared with matched controls without a placement.

**Meaning:**

These findings suggest that provision of noncongregate shelter with supportive services in SIP hotels during the COVID-19 pandemic was associated with reduced use of acute health services among people with prior high use.

## Teaser

This cohort study evaluates the association of shelter-in-place (SIP) hotel placements during the COVID-19 pandemic with health services use among a subset of people experiencing homelessness with prior high acute health service use.

## Introduction

People experiencing homelessness (PEH) have disproportionately high emergency department (ED) use, and this has held true during the COVID-19 pandemic.^1,2,3,4,5,6^ ED visits among the general population decreased by 42% nationally during the first months of the pandemic^7^ and remained 23% below prepandemic levels by November 2020.^8,9^ Despite this decrease in ED use among the general population, use among PEH remained close to prepandemic rates in some jurisdictions.^6^ Because of limited public health resources, many regions reduced existing outpatient health, mental health, and substance use treatment services and diverted efforts to COVID-19 diagnosis and treatment. Although many patients leveraged telehealth during the pandemic, PEH’s unstable living situations and lack of consistent access to telephones or computers may have contributed to continued barriers to accessing nonemergency care.^10^

PEH are at increased risk of COVID-19 infection and related morbidity and mortality,^11,12,13,14^ in part because of high transmission rates in congregate homeless shelters.^15^ People who are older or who have comorbidities are at higher risk for poor outcomes with COVID-19; many PEH meet these criteria.^16,17^ PEH do not have options for isolation or quarantine, which could lead to increased transmission and downstream outcomes, including the need for hospitalization.^18^

In April 2020, the City and County of San Francisco, California, implemented an emergency, shelter-in-place (SIP) hotel program to provide noncongregate shelter to PEH who were considered vulnerable to severe COVID-19 because of their age and/or comorbidities.^19^ As the result of federal policy allowing states to use Federal Emergency Management Agency funds to place PEH in noncongregate settings during the pandemic, localities have repurposed hotels as alternatives to high-density shelters. Beyond preventing COVID-19 transmission,^20^ little is known about other benefits of using hotels as noncongregate shelters. Other interventions based on housing-first principles, such as permanent supportive housing, have been shown to decrease use of emergency services.^21,22,23,24,25,26^ Some studies of permanent supportive housing have shown large reductions in emergency service use among those with prior high use,^27^ although other programs for frequent ED users have shown mixed results.^28,29^ Furthermore, medical respite programs—shelter and supportive services for PEH with medical need—have been shown to reduce use of hospital services, although less is known about the effects of these programs on PEH with prior high use.^30,31^ The rapid implementation of San Francisco’s SIP hotel program provides an opportunity to examine whether placement in noncongregate shelter with supportive services was associated with changes in service utilization for PEH who had been high users of acute health services.

## Methods

### Study Design and Setting

To reduce the transmission of COVID-19 among PEH, the City and County of San Francisco established SIP sites at 25 hotels, ranging from 30 to 450 beds, serving up to 2500 individuals at a time.^19^ To understand the impact of the program on those with high use of acute health services, we focused our analysis on the subset of SIP guests and their matched controls who were identified as the top 10% high users of multiple acute medical and behavioral health services in San Francisco and had 3 or more ED visits in 9 months. We used a matched retrospective cohort design to compare health services use among PEH with prior high use of acute health services who were placed in SIP hotels (intervention) and those who were not (control). We used negative binomial regression to compare changes in service utilization across intervention and control groups for 90 days before and 90 days after SIP placements occurring between April 2020 and April 2021. The University of California, San Francisco, institutional review board approved this research. Our report follows the Strengthening the Reporting of Observational Studies in Epidemiology (STROBE) reporting guideline for cohort studies. Informed consent was not provided because the study did not involve contact with individuals, in accordance with 45 CFR §46.

### Participants and Eligibility

Individuals were eligible for placement in a SIP hotel if they were experiencing homelessness and met the Centers for Disease Control and Prevention’s definition of high risk for severe COVID-19 based on age and underlying conditions.^32^ SIP programs defined individuals as experiencing homelessness if they lived outside or in a location not meant for human habitation, or were staying in the shelter system or another facility (eg, hospital, treatment facility, or jail) and had no other place to stay.

We included all SIP guests who were in the top 10% of high utilizers of multiple systems (HUMS) and used the ED 3 times or more during the eligibility period and a group of matched controls who were not placed in SIP hotels. Although all SIP guests were considered at risk for poor COVID-19 outcomes, those with high service use have greater disease burdens than the general PEH population.^16^ We assessed service utilization during the 9-month period before the implementation of the SIP hotel program from July 1, 2019, to March 31, 2020. The San Francisco Department of Public Health (SFDPH) assigns HUMS scores according to use of 9 emergency services across the medical, mental health, and substance use disorder systems, including ED visits, inpatient stays, psychiatric ED visits, and detoxification services.^33,34^ The HUMS score is an unweighted sum of these 9 urgent-emergent services. SFDPH uses HUMS scores to identify individuals experiencing fragmented care across multiple systems who would benefit from improved care coordination.

### Intervention

The SIP hotels provided private rooms, bathrooms, and 3 meals per day. Guests had access to on-site health care services, although availability varied by site. Each SIP hotel offered a minimum of 1 half-day nursing clinic and 1 half-day medical clinic per week and, at maximum, offered 40 hours per week of medical staffing at hotels geared toward guests with higher medical needs. The on-site medical clinics offered basic evaluations, treatment of minor conditions (eg, basic wound care), buprenorphine induction, and referrals and transportation to off-site clinics for full-scope primary and specialty care. Community clinics offered limited, prioritized scheduling for primary care appointments for individuals referred from a SIP hotel. Each SIP hotel had a housing navigator who assisted guests in transitioning from SIP hotels to permanent housing. Community-based organizations offered additional services (eg, naloxone, needle exchange, and benefits enrollment), although availability differed by site.

### Data Sources

We used administrative data from the Coordinated Care Management System (CCMS), an integrated database managed by the SFDPH that links information at the person-level from multiple county agencies.^16,33^ CCMS includes medical, behavioral health, and social service delivery data, as well as information on social needs including homelessness and shelter use. CCMS creates a record for anyone who self-reports as homeless in a health care encounter, uses homelessness services (eg, shelter or housing navigation), uses county behavioral health or emergent-urgent health services, or is booked in the county jail.^16^

### Measures

To describe the characteristics of the intervention and control groups at baseline, we used measures of age, gender, race and ethnicity, years homeless, in the top 5% and top 1% HUMS, and receipt of Social Security Income. Race and ethnicity were extracted from the CCMS records. We also included baseline measures of urgent care visits, outpatient medical visits, and all service use outcomes of interest. For outcome measures, we extracted encounter data on ED visits, inpatient stays and bed days, psychiatric ED visits, inpatient psychiatric stays, outpatient medical visits, outpatient mental health visit, and number of methadone or buprenorphine treatment visits.

### Statistical Analysis

We extracted records of individuals in the CCMS with emergency service use between July 1, 2019, and March 31, 2020 (45 473 individuals) and kept those with a HUMS score in the top 10th percentile (4665 individuals) (Figure). We excluded anyone without 3 or more ED visits in the eligibility period, leaving 3445 people. We then excluded people placed in nonhotel SIP sites including trailers, safe sleep sites for tents, and reduced-capacity congregate shelters. We isolated the subset of these high users who were placed in SIP hotels between April 2020 and April 2021 (440 individuals). We excluded those who were in SIP hotels for short stays (<90 days), resulting in 343 eligible people in the intervention group.

**Figure.**
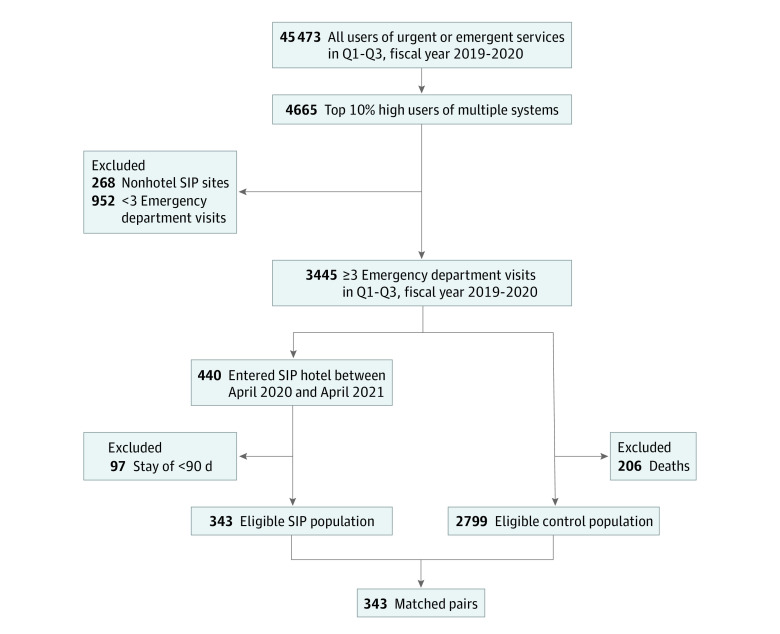
Study Enrollment Flowchart SIP indicates shelter-in-place; Q, quarter.

To create a matched control group, we isolated 3005 eligible controls who were the top 10% HUMS and had 3 or more ED visits during the 9-month eligibility period but were not placed in SIP hotels. We excluded those who died during the study period (206 individuals) leaving 2799. From these, we identified 343 controls, matched on demographics, homelessness status, and prestudy medical and behavioral health use. We used the GenMatch package to create a control group^35^ with a distribution of covariates similar to that of the SIP hotel cohort. We chose matched controls without replacement and used a 1-control-per-intervention strategy to optimize the similarity of the covariate distributions across the 2 groups. We compared baseline characteristics across the groups using χ^2^ tests (Fisher exact χ^2^ where cell counts were <5) for categorical measures and the Kruskal-Wallis test for continuous measures (Table 1).

**Table 1.** Sample Characteristics

Characteristic	Participants, No. (%)			*P* value^a^
	Total (N = 686 [100%])	Control (n = 343 [50%])	SIP hotel guest (n = 343 [50%])	
Age range, y				
18-24	6 (0.9)	3 (0.9)	3 (0.9)	.97
25-30	31 (4.5)	15 (4.4)	16 (4.7)	
31-40	107 (15.6)	57 (16.6)	50 (14.6)	
41-50	131 (19.1)	65 (19.0)	66 (19.2)	
51-60	231 (33.7)	117 (34.1)	114 (33.2)	
≥60	180 (26.2)	86 (25.1)	94 (27.4)	
Gender				
Women	176 (25.7)	94 (27.4)	82 (23.9)	.40
Men	485 (70.7)	240 (70.0)	245 (71.4)	
Transgender	5 (0.7)	2 (0.6)	3 (0.9)	
Not stated	20 (2.9)	7 (2.0)	13 (3.8)	
Race and ethnicity				
African American or Black	283 (41.3)	144 (42.0)	139 (40.5)	.97
Asian or Pacific Islander	23 (3.4)	12 (3.5)	11 (3.2)	
Declined or not stated	67 (9.8)	33 (9.6)	34 (9.9)	
Latino or Latina	61 (8.9)	30 (8.7)	31 (9.0)	
Multiracial or other^b^	16 (2.3)	9 (2.6)	7 (2.0)	
American Indian	9 (1.3)	3 (0.9)	6 (1.7)	
White	227 (33.1)	112 (32.7)	115 (33.5)	
Years homeless				
Never homeless	15 (2.2)	8 (2.3)	7 (2.0)	>.99
<1	94 (13.7)	48 (14.0)	46 (13.4)	
1-4	117 (17.1)	57 (16.6)	60 (17.5)	
5-9	123 (17.9)	61 (17.8)	62 (18.1)	
≥10	337 (49.1)	169 (49.3)	168 (49.0)	
Urgent or emergent services, No.				
<10	394 (57.4)	201 (58.6)	193 (56.3)	.81
11-20	189 (27.6)	91 (26.5)	98 (28.6)	
≥21	103 (15.0)	51 (14.9)	52 (15.2)	
HUMS score, median (IQR)	9 (6-15)	9 (6-15)	9 (6-16)	.71
In HUMS top 5%				
No	265 (38.6)	131 (38.2)	134 (39.1)	.81
Yes	421 (61.4)	212 (61.8)	209 (60.9)	
In HUMS top 1%				
No	555 (80.9)	278 (81.0)	277 (80.8)	.92
Yes	131 (19.1)	65 (19.0)	66 (19.2)	
Receives Social Security Income or Social Security Disability Income				
No	375 (54.7)	182 (53.1)	193 (56.3)	.40
Yes	311 (45.3)	161 (46.9)	150 (43.7)	
Emergency department visits, No.				
3-9	518 (75.5)	257 (74.9)	261 (76.1)	.96
10-19	104 (15.2)	52 (15.2)	52 (15.2)	
20-39	45 (6.6)	24 (7.0)	21 (6.1)	
≥40	19 (2.8)	10 (2.9)	9 (2.6)	
Psychiatric emergency visits, No.				
0	526 (76.7)	263 (76.7)	263 (76.7)	>.99
1	53 (7.7)	26 (7.6)	27 (7.9)	
2	60 (8.7)	30 (8.7)	30 (8.7)	
3-9	33 (4.8)	17 (5.0)	16 (4.7)	
10-19	11 (1.6)	6 (1.7)	5 (1.5)	
≥20	3 (0.4)	1 (0.3)	2 (0.6)	
Inpatient stays, No.				
0	318 (46.4)	164 (47.8)	154 (44.9)	.74
1	151 (22.0)	73 (21.3)	78 (22.7)	
≥2	217 (31.6)	106 (30.9)	111 (32.4)	
Urgent care visits, No.				
0	338 (49.3)	176 (51.3)	162 (47.2)	.15
1	156 (22.7)	66 (19.2)	90 (26.2)	
2-9	178 (25.9)	95 (27.7)	83 (24.2)	
≥10	14 (2.0)	6 (1.7)	8 (2.3)	
Outpatient medical visits, No.				
0	293 (42.7)	146 (42.6)	147 (42.9)	.64
1	85 (12.4)	45 (13.1)	40 (11.7)	
2-4	114 (16.6)	54 (15.7)	60 (17.5)	
5-15	142 (20.7)	76 (22.2)	66 (19.2)	
≥16	52 (7.6)	22 (6.4)	30 (8.7)	
Outpatient mental health visits, No.				
0	505 (73.6)	237 (69.1)	268 (78.1)	.09
1	17 (2.5)	9 (2.6)	8 (2.3)	
2	15 (2.2)	7 (2.0)	8 (2.3)	
3-9	39 (5.7)	20 (5.8)	19 (5.5)	
10-19	38 (5.5)	23 (6.7)	15 (4.4)	
20-39	27 (3.9)	19 (5.5)	8 (2.3)	
≥40	45 (6.6)	28 (8.2)	17 (5.0)	
Methadone maintenance and/or buprenorphine treatment visits, No.				
0	579 (84.4)	290 (84.5)	289 (84.3)	.82
1	10 (1.5)	4 (1.2)	6 (1.7)	
16-75	20 (2.9)	8 (2.3)	12 (3.5)	
76-200	40 (5.8)	21 (6.1)	19 (5.5)	
≥200	37 (5.4)	20 (5.8)	17 (5.0)	
Outpatient substance use treatment visits, No.				
0	654 (95.3)	326 (95.0)	328 (95.6)	.91
1-15	22 (3.2)	12 (3.5)	10 (2.9)	
≥16	10 (1.5)	5 (1.5)	5 (1.5)	

Abbreviations: HUMS, high utilizers of multiple systems; SIP, shelter-in-place.

^a^ *P* values were calculated using χ^2^ tests for categorical measures and the Kruskal-Wallis test for continuous measures.

^b^ Other was included as a category in the extracted record, with no other races or ethnicities specified.

After constructing the matched control group, we applied a parametric model to measure the impact of placement in a SIP hotel. This match-then-model approach aims to make parametric models produce more accurate and less model-dependent causal inferences.^36^ During the early months of the pandemic, there was lower use of EDs overall. To model these ED visits, we used a negative binomial regression model. As all outcomes were counts with a right-skewed distribution, we chose to analyze using negative binomial mixed models with random intercepts for each subject. We compared mean utilization at 2 time points: before and after SIP entry. Mean utilization was derived from visit-level utilization history, averaging visit counts across the 90-day period before SIP entry (baseline) and in the 90-day period after SIP entry (3 months). We calculated effect sizes to examine mean differences across SIP and non-SIP groups at baseline and 3 months and determined that mean differences at baseline were in the very small range (Cohen *d* = 0.02-0.18) for all outcome measures except outpatient visits (Cohen *d* = 0.21) (Table 2). We compared the experimental conditions across time by testing the group-by-time interaction effects using mixed-effects models. Fixed effects included in all models were intervention group assignment, time, and their interaction, with both group and time effects treated as categorical variables. We estimated mixed-effects models using maximum likelihood, which we specified to contain random intercepts with an unstructured covariance matrix. Significance was set at *P* < .05 (2-tailed). We used Stata statistical software version 17 (StataCorp) to perform the analyses. We performed a sensitivity analysis on a smaller subgroup of SIP hotel guests who stayed 180 days or longer and their matched controls to examine utilization in the 180 days before and after SIP hotel placement. Data analysis for this study was performed from February 2021 to May 2022.

**Table 2.** Effect Sizes at Baseline and 90 Days After SIP Entry for SIP Guests and Matched Controls

Type of service	Mean (95% CI)		Cohen *d* (95% CI)^a^
	SIP hotel guest (n = 343)	Control (n = 343)	
Emergency department visits, No.			
Baseline	1.84 (1.52 to 2.17)	1.33 (1.39 to 1.58)	0.05 (–0.20 to 0.10)
3 mo	0.82 (0.66 to 0.99)	1.00 (0.80 to 1.20)	0.20 (0.04 to 0.34)
Inpatient days, No.			
Baseline	4.00 (2.44 to 5.56)	2.27 (1.27 to 3.27)	0.17 (0.02 to 0.32)
3 mo	0.81 (0.40 to 1.23)	1.85 (1.06 to 2.65)	0.20 (0.05 to 0.35)
Inpatient stays, No.			
Baseline	0.41 (0.30 to 0.51)	0.27 (0.19 to 0.34)	0.18 (0.03 to 0.33)
3 mo	0.14 (0.09 to 0.19)	0.22 (0.15 to 0.29)	0.18 (0.03 to 0.33)
Psychiatric emergency visits, No.			
Baseline	0.03 (0.01 to 0.05)	0.02 (0.01 to 0.04)	0.08 (–0.07 to 0.23)
3 mo	0.01 (0.00 to 0.01)	0.02 (0.00 to 0.03)	0.18 (0.03 to 0.33)
Inpatient psychiatric stays, No.			
Baseline	0.00 (0.00 to 0.01)	0.00 (0.00 to 0.01)	0.05 (–0.10 to 0.20)
3 mo	0.00 (0.00 to 0.00)	0.00 (0.00 to 0.00)	0.06 (–0.09 to 0.21)
Outpatient visits, No.			
Baseline	0.45 (0.31 to 0.59)	0.25 (0.17 to 0.34)	0.21 (0.06 to 0.36)
3 mo	0.33 (0.22 to 0.45)	0.24 (0.15 to 0.32)	0.07 (–0.08 to 0.22)
Outpatient mental health visits, No.			
Baseline	2.32 (1.24 to 3.40)	2.81 (1.52 to 4.11)	0.06 (–0.09 to 0.21)
3 mo	2.88 (1.47 to 4.29)	3.05 (1.56 to 4.54)	0.02 (–0.13 to 0.17)
Methadone maintenance and/or buprenorphine treatment visits, No.			
Baseline	6.64 (1.93 to 11.36)	6.94 (2.02 to 11.87)	0.02 (–0.13 to 0.16)
3 mo	8.96 (2.42 to 15.51)	7.89 (2.12 to 13.67)	0.04 (–0.11 to 0.19)

Abbreviation: SIP, shelter-in-place.

^a^ Standardized difference *d* (Cohen *d*) interpretation: If the 95% CI spans 0, there is no difference in means (*d* < 0.2, very small effect; 0.2 ≤ *d* <0.5, small effect; 0.5 ≤ *d* <0.8, medium effect; and *d* ≥ 0.8, large effect).

## Results

After matching, we identified 686 (343 SIP intervention; 343 control) PEH who had 3 or more ED visits with a HUMS score in the top 10%. The 343 SIP guests who met criteria for our study comprised 13.6% of all 2524 guests who stayed in SIP for at least 90 days. Participants spent a mean (SD) of 301.0 (116.8) days in a hotel. Table 1 shows the demographics and characteristics of the matched study sample. The study sample consisted of participants aged 21 to 88 years (median [IQR], 54 [43-61] years), with 485 (70.7%) self-identifying as male, 283 (41.3%) as Black, 227 (33.1%) as White, and 61 (8.9%) as Latino or Latina. Approximately one-half (337 participants [49.1%]) were homeless for more than 10 years. In the 9-month period before the SIP hotel implementation, 518 (75.5%) had 3 to 9 ED visits and 104 (15.2%) had 10 to 19 ED visits. More than one-half (368 participants [53.6%]) had 1 or more hospitalization. The median (IQR) HUMS score was 9 (6-15). We found no significant differences between the intervention and matched control groups for any covariates.

 The majority (54% [185 individuals]) of the total SIP guests with a minimum of 90-day stays had no acute service use during the eligibility period. ED visits decreased in the intervention group from a mean of 1.84 visits (95% CI, 1.52-2.17 visits) before the SIP intervention to 0.82 visits (95% CI, 0.66-0.99 visits) in the 90 days after the intervention, a 55.4% decrease, compared with a 24.8% reduction in the control group from 1.33 (95% CI, 1.39-1.58 visits) to 1.00 (95% CI, 0.80-1.20 visits) (incidence risk ratio [IRR], 0.60; 95% CI, 0.47-0.75; *P* < .001), representing a 40% reduction in ED visits relative to the control group (Table 3). The number of hospitalizations decreased from a mean of 0.41 (95% CI, 0.30-0.51) to 0.14 (95% CI, 0.09-0.19) for the intervention group and from 0.27 (95% CI, 0.19-0.34) to 0.22 (95% CI, 0.15-0.29) visits for the control group (IRR, 0.41; 95% CI, 0.27-063; *P* < .001), a 59% reduction for the intervention group relative to the control group. Inpatient hospital days decreased by 75% in the intervention group relative to the control group, from a mean of 4.00 (95% CI, 2.44-5.56) to 0.81 (95% CI, 0.40-1.23), a 79.8% decrease compared with an 18.5% decrease from 2.27 (95% CI, 1.27-3.27) to 1.85 (95% CI, 1.06-2.65) among controls (IRR, 0.25; 95% CI, 0.12-0.54; *P* < .001). Psychiatric ED visits among those placed in SIP hotels decreased by 75% relative to the control group, from a mean of 0.03 (95% CI, 0.01-0.05) to 0.01 (95% CI, 0.00-0.01) compared with no change among controls (IRR, 0.25; 95% CI, 0.11-0.51; *P* < .001). We found no difference in outpatient medical visits (IRR, 0.80; 95% CI, 0.56, 1.15; *P* = .24), in-patient psychiatric stays (IRR, 1.18; 95% CI, 0.26, 5.46; *P* = .83), outpatient mental health visits (IRR, 1.15; 95% CI, 0.46, 2.85; *P* = .77), or number of methadone or buprenorphine treatment visits (IRR, 1.19; 95% CI, 0.30, 4.76; *P* = .81). In the sensitivity analysis looking at 180-day time spans, we found similar results as in the primary analysis, with a significant postintervention reduction in ED visits, hospital length of stay, and psychiatric ED visits when compared with matched controls (Table 4).

**Table 3.** Comparison of Total Health Care Service Utilization 90 Days Before and After SIP Hotel Placement

Type of service	Mean (95% CI), No.^a^				IRR (95% CI)^b^	*P* value
	SIP hotel placement		Control			
	Before	After	Before	After		
Emergency department visits	1.84 (1.52-2.17)	0.82 (0.66-0.99)	1.33 (1.39-1.58)	1.00 (0.80-1.20)	0.60 (0.47-0.75)	<.001
Inpatient days	4.00 (2.44-5.56)	0.81 (0.40-1.23)	2.27 (1.27-3.27)	1.85 (1.06-2.65)	0.25 (0.12-0.54)	<.001
Inpatient stays	0.41 (0.30-0.51)	0.14 (0.09-0.19)	0.27 (0.19-0.34)	0.22 (0.15-0.29)	0.41 (0.27-0.63)	<.001
Psychiatric emergency visits	0.03 (0.01-0.05)	0.01 (0.00-0.01)	0.02 (0.01-0.04)	0.02 (0.00-0.03)	0.25 (0.11-0.51)	<.001
Inpatient psychiatric stays	0.00 (0.00-0.01)	0.00 (0.00-0.00)	0.00 (0.00-0.01)	0.00 (0.00-0.00)	1.18 (0.26-5.46)	.83
Outpatient medical visits	0.45 (0.31-0.59)	0.33 (0.22-0.45)	0.25 (0.17-0.34)	0.24 (0.15-0.32)	0.80 (0.56-1.15)	.24
Outpatient mental health visits	2.32 (1.24-3.40)	2.88 (1.47-4.29)	2.81 (1.52-4.11)	3.05 (1.56-4.54)	1.15 (0.46-2.85)	.77
Methadone and/or buprenorphine treatment visits	6.64 (1.93-11.36)	8.96 (2.42-15.51)	6.94 (2.02-11.87)	7.89 (2.12-13.67)	1.19 (0.30-4.76)	.81

Abbreviations: IRR, incidence rate ratio; SIP, shelter-in-place.

^a^ Estimated mean and 95% CI were calculated by negative binomial regression.

^b^ IRRs and 95% CIs were calculated using negative binomial regression models. The IRR represents the utilization rate in the intervention group compared with the matched control group.

**Table 4.** Comparison of Health Care Service Utilization 180 Days Before and After SIP Hotel Placement

Type of service	Mean (95% CI), No.^a^				IRR (95% CI)^b^	*P* value
	SIP hotel placement		Control			
	Before	After	Before	After		
Emergency department visits	4.36 (3.43 to 5.30)	1.56 (1.18 to 1.95)	3.58 (2.81 to 4.36)	2.10 (1.59 to 2.61)	0.61 (0.49 to 0.76)	<.001
Inpatient days	8.07 (4.38 to 11.77)	1.92 (0.73 to 3.11)	6.82 (3.55 to 10.08)	3.38 (1.64 to 5.11)	0.48 (0.24 to 0.95)	.03
Inpatient stays	0.63 (0.43 to 0.83)	0.30 (0.19 to 0.42)	0.58 (0.40 to 0.76)	0.38 (0.25 to 0.52)	0.73 (0.50 to 1.06)	.10
Psychiatric emergency visits	0.05 (0.00 to 0.10)	0.00 (0.00 to 0.01)	0.04 (0.00 to 0.07)	0.01 (0.00 to 0.03)	0.25 (0.11 to 0.53)	<.001
Inpatient psychiatric stays	0.01 (0.00 to 0.02)	0.00 (0.00 to 0.01)	0.01 (0.00 to 0.03)	0.00 (0.00 to 0.01)	1.24 (0.30 to 5.20)	.77
Outpatient medical visits	1.03 (0.67 to 1.40)	0.76 (0.41 to 1.11)	0.64 (0.40 to 0.88)	0.55 (0.30 to 0.81)	0.85 (0.59 to 1.22)	.38
Outpatient mental health visits	3.62 (1.45 to 5.79)	5.32 (1.40 to 9.25)	5.26 (1.95 to 8.57)	5.61 (1.56 to 9.67)	1.38 (0.50 to 3.82)	.54
Methadone and/or buprenorphine treatment visits	15.06 (1.41 to 28.71)	19.96 (–1.60 to 41.52)	14.11 (0.43 to 27.80)	16.49 (–0.22 to 33.19)	1.13 (0.24 to 5.37)	.87

Abbreviations: IRR, incidence rate ratio; SIP, shelter-in-place.

^a^ Estimated mean and 95% CI were calculated by negative binomial regression.

^b^ IRRs and 95% CIs were calculated using negative binomial regression models. The IRR represents the utilization rate in the intervention group compared with the matched control group.

## Discussion

In a matched retrospective cohort study of PEH with prior high use of acute health services, we found that people who spent at least 90 days in SIP hotels had significant decreases in acute health services use compared with similar people who did not receive a SIP hotel placement. Our findings support our hypothesis that noncongregate shelter combined with on-site supportive services can reduce use of acute health services among PEH with prior high use.

For study participants who did not receive a SIP hotel placement, ED visits decreased by 24.8% during the pandemic. This was similar to the 23% decrease in ED use seen in the general population,^8^ but much smaller than the 55.4% reduction seen in the group who received a SIP placement. Other housing interventions, such as permanent supportive housing, have not shown a similar association with ED visits for PEH with prior high use of acute health services,^29^ although there is some evidence these programs may reduce acute care use for the general PEH population.^25,26,37^

PEH have longer hospital lengths of stay, because of the difficulty of identifying safe discharge options. Here, SIP placement had a substantial impact on length of stay, decreasing inpatient days by 79.8% over the study period, whereas the length of stay for controls decreased by 18.5%. Our findings are consistent with previous research^31^ demonstrating that access to medical respite—that is, shelter with medical services—reduces inpatient days for PEH. In addition, compared with matched controls, participants who received a SIP hotel placement had significantly fewer hospitalizations and psychiatric ED visits.

These findings provide evidence that using hotels as noncongregate shelters can benefit PEH who are high users of acute health services, beyond preventing outbreaks of COVID-19. SIP hotel placement in Washington State was associated with improvements in self-reported health, increased sense of safety, and reduced conflicts and 911 calls.^38^ The SIP hotels in San Francisco had varying degrees of embedded health services, as well as increased access to local clinics. These services may have contributed to the reductions in acute health services use by meeting the needs of this high user population outside of the hospital system. It may also be that the provision of temporary noncongregate shelter prevented some acute problems experienced by PEH, such as exposure-related illness, violence, or public intoxication, that can result in ED visits or hospital admissions.

We found significant differences in acute health services use in a relatively short 90-day period for the subset of PEH placed in SIP who were also frequent users of acute health services. Conversely, studies^29^ of the impact of permanent supportive provision among frequent health system users have shown it can be difficult to achieve these outcomes. Chronically homeless individuals who are eligible to receive permanent supportive housing placements may be more ill than our study population and already have heavy burdens of illness and high mortality rates, making acute health services use difficult to reduce or avoid. In addition, few permanent supportive housing programs include the level of embedded on-site health care offered by the SIP sites, which may have contributed to our findings.

The implementation of SIP hotel programs throughout the US demonstrates the possibility of rapidly transforming unused or underused locations such as hotels for noncongregate shelter. However, SIP hotels were funded by Federal Emergency Management Agency and were intended to be a temporary emergency measure. Further research and policy making should continue to focus on increasing the supply of permanent supportive housing, and affordable housing for extremely low-income households, as well as sustainable models of noncongregate shelter where needed.

### Limitations

We examined health service use among a subset of SIP hotel guests who were high users of acute health services, and our findings may not apply to the broader SIP guest or PEH populations. The majority (54%) of the total SIP guests with a minimum of 90-day stays had no acute service use during the eligibility period. Furthermore, regression to the mean can bias studies of high users of health services. Although we used a matched control group to mitigate this issue, by focusing on the high user population, we selected for participants who would be most likely to show changes in acute service use.

We evaluated our outcomes over 90 days only. Except for inpatient stays, our results also held over a 180-day period, but more research is needed to verify the durability of our findings. Observational studies are a lower standard of evidence than experimental studies, are more prone to bias and confounding, and cannot be used to demonstrate causality. We mitigated these limitations by using a matched control group. People who consented to enter SIP hotels may differ from controls in ways we did not measure. The intervention was based in San Francisco County, and our findings may not be generalizable to other jurisdictions.

## Conclusions

In a population of PEH with high use of multiple health services, SIP hotel placement during the COVID-19 pandemic was associated with significantly reduced acute health service use compared with high users without a placement. Using existing hotels as noncongregate shelter with embedded health services may be an effective strategy to mitigate COVID-19 risks as well as to reduce acute care use among PEH with a history of high health services use.
